# A mast‐seeding desert shrub regulates population dynamics and behavior of its heteromyid dispersers

**DOI:** 10.1002/ece3.2035

**Published:** 2016-03-04

**Authors:** Janene Auger, Susan E. Meyer, Stephen H. Jenkins

**Affiliations:** ^1^ Program in Ecology, Evolution and Conservation BiologyUniversity of NevadaRenoNevada89557; ^2^ USFS Rocky Mountain Research StationShrub Sciences LaboratoryProvoUtah84606; ^3^ Department of BiologyUniversity of NevadaRenoNevada89557; ^4^ Present address: Monte L Bean Life Science MuseumBrigham Young UniversityProvoUtah84602

**Keywords:** Blackbrush, *Coleogyne ramosissima*, *Dipodomys ordii*, interference competition, kangaroo rat, optimal caching theory, *Perognathus flavescens*, pocket mouse, resource abundance hypothesis, scatterhoarding

## Abstract

Granivorous rodent populations in deserts are primarily regulated through precipitation‐driven resource pulses rather than pulses associated with mast‐seeding, a pattern more common in mesic habitats. We studied heteromyid responses to mast‐seeding in the desert shrub blackbrush (*Coleogyne ramosissima*), a regionally dominant species in the Mojave–Great Basin Desert transition zone. In a 5‐year study at Arches National Park, Utah, USA, we quantified spatiotemporal variation in seed resources in mast and intermast years in blackbrush‐dominated and mixed desert vegetation and measured responses of *Dipodomys ordii* (Ord's kangaroo rat) and *Perognathus flavescens* (plains pocket mouse). In blackbrush‐dominated vegetation, blackbrush seeds comprised >79% of seed production in a mast year, but 0% in the first postmast year. Kangaroo rat abundance in blackbrush‐dominated vegetation was highest in the mast year, declined sharply at the end of the first postmast summer, and then remained at low levels for 3 years. Pocket mouse abundance was not as strongly associated with blackbrush seed production. In mixed desert vegetation, kangaroo rat abundance was higher and more uniform through time. Kangaroo rats excluded the smaller pocket mice from resource‐rich patches including a pipeline disturbance and also moved their home range centers closer to this disturbance in a year of low blackbrush seed production. Home range size for kangaroo rats was unrelated to seed resource density in the mast year, but resource‐poor home ranges were larger (*P *<* *0.001) in the first postmast year, when resources were limiting. Blackbrush seeds are higher in protein and fat but lower in carbohydrates than the more highly preferred seeds of Indian ricegrass (*Achnatherum hymenoides*) and have similar energy value per unit of handling time. Kangaroo rats cached seeds of these two species in similar spatial configurations, implying that they were equally valued as stored food resources. Blackbrush mast is a key resource regulating populations of kangaroo rats in this ecosystem.

## Introduction

**Figure 1:**
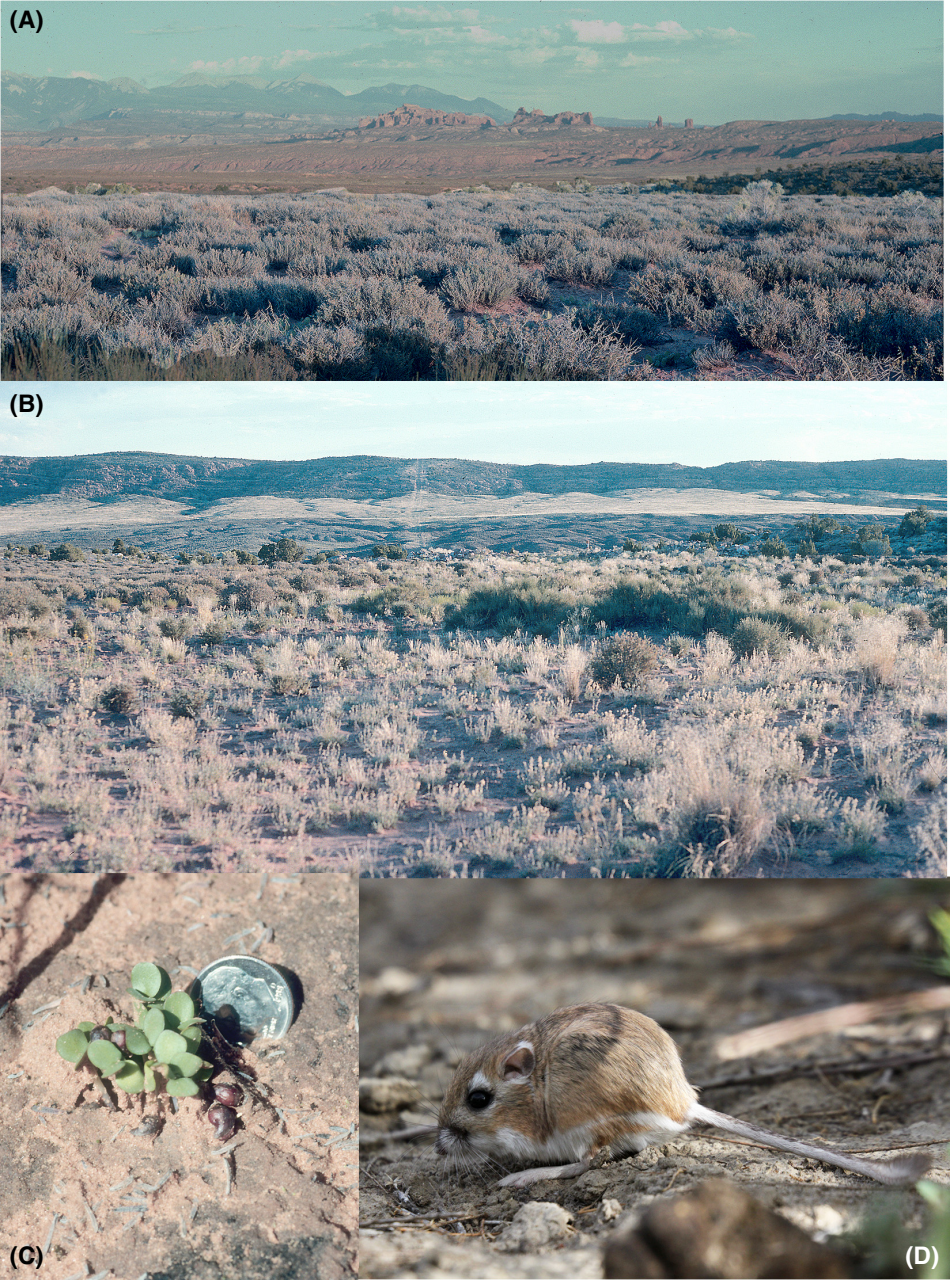
(A) Blackbrush‐dominated vegetation at Salt Valley, (B) herbaceous vegetation on the pipeline corridor, Salt Valley, (C) blackbrush seedlings emerging from a scatterhoard, and (D) Ord's kangaroo rat (*Dipodomys ordii*; photograph: Lindsay Dimitri; other photographs by authors).

Populations of granivorous rodents in desert environments encounter dramatic within‐ and between‐year variability in available food resources. Amount and timing of precipitation are usually the driving force for seed production of annual plants and most perennials (Beatley 1974). However, seed resource availability may be uncoupled from precipitation patterns if the vegetation is dominated by a mast‐seeding species. Mast‐seeding is defined as high and synchronous interannual variation in seed production as an evolutionary response to selection related to economies of scale, for example, increased probability of escape from seed predation in years of high production (“normal” masting; Kelly 1994). It is a common phenomenon in mesic environments but has rarely been reported in deserts (Kelly and Sork 2002). Blackbrush (*Coleogyne ramosissima*), a dominant Rosaceous shrub on three million hectares along the boundary between the Mojave and Great Basin deserts of North America (Bowns and West 1976), is apparently one of very few desert shrubs to exhibit “normal” masting (Meyer and Pendleton 2015a; Fig. 1A). Postdispersal seed predator satiation has been implicated as a primary selective force for masting in this species (Meyer and Pendleton 2005, 2015a,b).

A desert environment strongly dominated by a masting shrub should show temporal variation in availability of food resources that influences the behavior, population dynamics, and competitive interactions of granivorous rodents. Instead of directly tracking primary productivity driven by variation in precipitation (Previtali et al. 2009; Thibault et al. 2010), patterns of rodent response are expected to track temporal patterns in seed availability that result from resource pulses generated by the cycle of mast and intermast years (Schmidt and Ostfeld 2008).

In North American deserts, the predictability of rodent population fluctuations is further complicated by the evolved food‐hoarding behaviors of heteromyids, the primary granivorous rodents in these communities (Vander Wall 1990). Scatterhoarding (placement of seeds in cryptic caches just under the soil surface) may also have positive fitness consequences for the masting species, as these rodents are also important as seed dispersal agents (Fig. 1C; Vander Wall 2010). We tested the hypothesis that heteromyid abundance would track blackbrush masting patterns by characterizing rodent populations over time using trapping data in both blackbrush‐dominated vegetation and vegetation dominated by nonmasting species.

Our study focused primarily on the resident Ord's kangaroo rat (*Dipodomys ordii*; Fig. 1D), but we also examined interactions between *D. ordii* and the only other resident heteromyid at our study site, the plains pocket mouse (*Perognathus flavescens*). We used trapping data to evaluate niche overlap and habitat use by the two species in the context of spatial and temporal variation in seed resources, which provided an indirect test of the hypothesis of competition for seed resources in resource‐rich patches via interference competition (Reichman and Price 1993).

Spatial variability of seed resources in deserts may also have a major influence on heteromyid movements and home range sizes. Factors influencing home range size and shape have received considerable theoretical attention (Adams 2001; Prevedello et al. 2013). The fundamental prediction is that home range area should decrease with increasing food mass per unit area (i.e., the resource abundance hypothesis; Akbar and Gorman 1993; Lacher and Mares 1996). We tested this hypothesis in an unmanipulated setting in which natural spatial and temporal variation in food resources was quantified. We accounted for differences in food resources available to individuals due to the relative proportions of different habitat types with different levels of resource availability within each home range and related seed availability expressed as seed mass per unit area to home range size in years of contrasting resource abundance, namely mast and intermast years.

We also examined the seed resources locally available to heteromyids in terms of kangaroo rat seed preference and its relationship to palatability, nutrient content, water balance, energetics, and seed handling time. We tested the hypothesis that seeds would be selected on the basis of maximization for energy, nutrients, or metabolic water content.

We selected two seed species for use in a field caching experiment to test the predictions of optimal caching theory, that is, to learn whether kangaroo rats would place caches of a preferred seed species in a spatial configuration less prone to pilferage by competitors than seeds of a less preferred seed species (Stapanian and Smith 1984; Vander Wall 1995; Steele et al. 1996; Leaver and Daly 1998). We predicted that more preferred seeds would be transported farther from the source, be placed in smaller, more widely spaced scatterhoards, and be more likely to be placed in home range core areas than areas of home range overlap. An alternative hypothesis was that seeds of the two species would be cached similarly, either because they were equally valued as stored seed resources in spite of a preference difference for immediate consumption, or because initial rapid sequestration close to the food source was the immediate behavioral response to the food bonanza represented by the bait station (Jenkins and Peters 1992).

## Materials and Methods

### Study site description

The field studies were conducted in Arches National Park, Grand Co., Utah, USA. Vegetation there consists of various assemblages of desert shrubs and small trees, succulents, grasses, and forbs that are regulated mostly by soil depth and the geology of the underlying landforms (Armstrong 1982). Arches National Park is in a relatively arid region, with an average precipitation of 227 mm per year, mostly in winter and as late summer monsoons (Western Regional Climate Center [Link]; Table 1). The average mean temperature is 13.8°C with average summer highs near 35°C and average winter highs near 7°C. Nighttime lows average 18°C during the summer and −6.5°C during the winter. The freeze‐free period averages 200 days.

**Table 1:** Precipitation at the weather station at park headquarters, Arches National Park (38°37′N, 109°37′W; elevation 1260 m) for the years 1997–2001 as well as 1980–2001 mean (Western Regional Climate Center [Link]). Monthly totals were summed from October through May (winter precipitation) and from June through September (summer precipitation)

Season	Precipitation (mm)					
	1997	1998	1999	2000	2001	1980–2001 mean
Winter	175	128	165	83	147	149
Summer	124	66	138	37	93	78

We conducted field studies at two sites, a primary blackbrush‐dominated site at Salt Valley and a secondary site at Willow Flats, where blackbrush is a minor component. The rodent communities at both sites included *Peromyscus maniculatus, Peromyscus truei, Onychomys leucogaster,* and *Ammospermophilus leucurus* in addition to the two heteromyid species mentioned earlier.

The 4‐ha Salt Valley study site (38°45′41″ N, 109°36′02″ W) has blackbrush‐dominated vegetation (Fig. 1A) on shallow soil over Entrada Sandstone bedrock, with somewhat different plant communities in wash bottoms, on exposed bedrock, and on a pipeline disturbance where sandy soils are deeper (Fig. 1B; Appendix 1). This site was used continuously through the study period from June 1997 through October 2001.

The 3.3‐ha Willow Flats study site (38°41′53″ N, 109°36′11″ W) is 7 km from the Salt Valley site. The area includes sand hummocks with deeper soils on the eastern side and flatter desert pavement with shallow cryptogamic soils on the western side. It is vegetated with mixed desert shrub grassland (Appendix 1). Studies at this site were initiated in late summer 1999, after kangaroo rat abundance at Salt Valley became too low to permit experimental studies.

### Habitat characterization

#### Habitat delineation

Habitat types for each study site were first digitized as polygon shapefiles in ArcView GIS 3.2 (ESRI 1999) from aerial photographs. At Salt Valley, the designated habitats were blackbrush, wash, rock outcrop, pipeline corridor, and road (no vegetation). At Willow Flats, the deeper soils of the sand hummocks on the east were separated from the shallow cryptogamic soils on the west by a relatively sharp ecotone.

#### Vegetation survey

We used 50 m × 2 m belt transects to survey vegetation during the growing season in each habitat to determine which plants were potentially significant as food sources for heteromyids. At Salt Valley, six transects were located in blackbrush, three on the pipeline corridor, three in wash habitat, and two in rock habitat. At Willow Flats, ten north–south transects were arranged systematically through the study site. Five were located on the east side (sandy hummocks) and five on the west side (shallow soils).

Twenty 1 m^2^ quadrats were randomly selected along each belt transect. Within each quadrat, individual shrubs, succulents, and perennials were counted by species and scored as juvenile or mature. Annuals were categorically ranked by species as low (≤10 individuals), medium (11–50 individuals), or high (50–100 individuals) in abundance. These data yielded percent frequency for all species and density for shrubs and perennials. Percent cover for woody plants was calculated using the line intercept method along each transect (Canfield 1941). Data from transects within the same habitat were pooled (Appendix 1).

### Seed resource quantification

Species that were significant components of each habitat were selected as potentially important to heteromyids based on the following criteria: density ≥0.10 mature individuals per m^2^, frequency >20%, or cover ≥5% (Appendices 1, 2).

#### Blackbrush

Blackbrush seed production was calculated in each habitat for each year based on three variables: shrub surface area per unit of habitat area, flowering intensity (an ordinal variable with four levels scored visually for each of >100 plants of reproductive size each year), and seed fill (filled seeds per flower measured each year; fruits are single‐seeded). Shrub surface area per unit of habitat area was obtained by measuring height and crown for all (>100) blackbrush plants of reproductive size within a belt transect of known area, estimating the surface area of each individual using the formula for a truncated cylinder (i.e., without the bottom that contacted the ground), summing the surface areas, and dividing by the transect area. Flowering intensity was converted to flowers per unit of shrub surface area using a regression equation developed by stripping and counting all the flowers on individual plants of known surface area for several plants in each flowering intensity category, while fill was obtained by examining all the flowers on a subset of ten branches each year to determine the proportion that contained a filled seed (Meyer and Pendleton 2015a). Blackbrush seed mass per unit area (SMPA in mg/m^2^) was then calculated for each combination of year and habitat using the following equation:

$$\text{SMPA}=\left(\sum_{i=0}^{3}A\cdot I_{i}\cdot F_{i}\cdot S\right)\cdot M$$

*A *= Shrub surface area per unit habitat area (constant over years, varies by habitat).

*I*_i_ = Proportion of shrubs in flowering intensity class *i* (varies by year).

*F*_i_ = Flowers per unit of shrub surface area in flowering intensity class *i* (regression equation, constant over years).

*S *= Seed fill proportion (varies by year).

*M *= Seed mass (assumed constant, 16.3 mg).

This direct method resulted in accurate estimates of blackbrush seed production each year.

#### Other shrubs, perennials, and annuals

To quantify viable seed production for other important shrubs and perennials, we collected and evaluated all seeds from 2 to 10 typical plants of each species, whereas for annual grasses and forbs, we collected from 10 to 25 whole plants (Appendix 2). Collections were made in fall 1999, spring 2000, and spring 2001; each plant species was collected in one of the 3 years. We estimated seed production for each species in other years by assuming a linear relationship between precipitation and seed production, using precipitation in a year with low or no seed production as the second point defining the line (Selås 2000; see Appendix 2 for details of calculation). We multiplied estimated seed production per plant by estimated plant density to obtain seed mass per unit area each year for each important species. For each habitat and year, the sum of SMPA for all important species, including blackbrush, comprised the total estimated SMPA.

### Trapping study

#### Trapping procedure

We established a permanent grid of 163 trapping stations at Salt Valley in summer 1997 and a grid of 156 stations at Willow Flats in summer 1999. Trap stations at both study sites were placed 15 m apart, and trap lines ran north to south. We live‐trapped rodents in Sherman traps (7.5 × 9.5 × 30.5 cm) using mixed birdseed and following the guidelines of the American Society of Mammalogists (1987). Traps were set in the evening and checked early the next morning. Captured animals were tagged in one or both ears with numbered metal tags, weighed, assessed for reproductive condition, and then released at the recorded point of capture. We were primarily interested in heteromyids but recorded all captures.

Trapping at Salt Valley occurred in 15 sessions from June 1997 to October 2001. A session consisted of either two nights (five sessions) or three nights (10 sessions). Trapping at Willow Flats occurred in 11 sessions from September 1999 to October 2001 (seven 2‐night sessions and four 3‐night sessions). We included both 2‐night and 3‐night sessions in our population estimates because we observed that heteromyids in our study were highly trappable, as has been confirmed by others (Price and Kelly 1994). We used minimum number known alive (MNKA) to track changes in rodent populations through time. MNKA can effectively show changes in population size over time and is useful in studies of environmental correlates of abundance, particularly when sample sizes are small (Boonstra et al. 1998). MNKA was strongly correlated with both closed and open rodent population estimators in an earlier study (*r* > 0.8; Slade and Blair 2000). The trapping data were also used for several secondary analyses as described below.

#### Habitat use and niche differentiation

To investigate possible competition between *D. ordii* and *P. flavescens* at Salt Valley, we first calculated the observed niche overlap for all species–time period combinations. The 163 trap stations were considered the common resource. We used the Czekanowski index (Feinsinger et al. 1981) as an estimate of niche overlap:

$$1.0-0.5\sum_{i=1}^{163}\left|p_{i}-r_{i}\right|,$$

where *p_i_* and *r*_*i*_ represent the proportions of captures at trap station *i* for *D. ordii* and *P. flavescens*, respectively.

We then used a randomization procedure with 1000 trials to generate a distribution of niche overlap matrices to which the observed values could be compared (True BASIC program, S. Jenkins). The procedure generated random numbers of captures at each trapping station for each species–time period combination while keeping fixed the values of (1) the total number of captures at each station over all species–period combinations and (2) the total number of captures for each species–period combination over all stations. We could then answer the question of whether there was more or less between‐species niche overlap observed within time periods (1997, 1998, and 1999–2001) than expected by chance by examining the probability of the occurrence of the observed niche overlap values relative to the distribution of values generated by the randomization procedure.

To address the question of whether *D. ordii* and *P. flavescens* used the resource‐rich pipeline area differentially, we used the Salt Valley trapping data to calculate the odds ratios of likelihood of capture on the pipeline corridor. For each species in each time period, the odds of being caught on the pipeline was (number of captures on the pipeline)/(number of captures off the pipeline), while the expected odds with no preference for or against the pipeline was (number of stations on the pipeline)/(number of stations off the pipeline). The odds ratio as an index of preference was the ratio of these two values, with a ratio >1 indicating more use than expected and a ratio <1 indicating less. We then analyzed the numbers of captures in a three‐way contingency table of species × time period × habitat. This used a log‐linear approach that allowed the stepwise testing of a series of nested models to screen the interaction terms for significance. Likelihood ratio chi‐square values (G) were used to test each model until the best fit (least difference between observed and expected values) was found (SPSS Science 1998).

### Home range study

#### Telemetry

We used radio telemetry to study movements of kangaroo rats in two periods at Salt Valley (24 June 1997–4 August 1997 and 4 July 1998–4 August 1998) and in one period at Willow Flats (25 October 1999–7 December 1999). We fitted kangaroo rats with either SM1 mouse‐style collar transmitters (AVM Instrument Company, Ltd., Livermore, CA) or MD‐2CT collar transmitters (Holohil Systems, Ltd., Carp, Ontario, Canada).

At Salt Valley, 14 kangaroo rats (six males and eight females) were collared in 1997 and 11 (five males and six females) were collared in 1998. A total of 634 free‐ranging locations were obtained as well as 88 day burrow locations. Data from regular trapping sessions confirmed that in both years, all resident adult kangaroo rats were collared, that is, the only uncollared individuals captured were juveniles or single captures, whereas collared animals were captured repeatedly. At Willow Flats, telemetry data were combined with trapping data obtained during the same time period (October–December 1999) to calculate home ranges. Telemetry (*n *=* *56) and trapping locations (*n *=* *126) were collected for all resident adult kangaroo rats (four males and six females).

#### Statistical analysis

For each individual with ≥8 free‐ranging locations (i.e., when the animal was out of its burrow), we used the program Animal Movement (Hooge et al. 1999; a 3rd‐party plug‐in for ArcView) to calculate a 95% fixed kernel home range utilization distribution (“home range”; Worton 1989) using the ad hoc calculation of a smoothing parameter (Silverman 1986). For each study site, we calculated home range sizes and overlaid the home ranges onto previously generated habitat maps to calculate the total area of each habitat in each home range each year. We then multiplied each habitat area in each home range by the total seed mass per unit area (SMPA in mg/m^2^) for the appropriate habitat and year, summed values across habitats, and divided by total area to obtain a weighted average of SMPA for each home range. To test the resource abundance hypothesis at Salt Valley, we used the home range data for the mast year (1997) and the first postmast year (1998) in separate analyses to examine the correlation between home range area and SMPA. One home range was eliminated from the 1998 correlation as an extreme outlier. We also examined the effect of year on home range size and SMPA at Salt Valley using ANOVA.

At Salt Valley, we predicted that kangaroo rats should move closer to the resource‐rich pipeline habitat during times of resource scarcity. We measured this effect by averaging the unique burrow locations of each kangaroo rat home range to obtain a center and then calculating a perpendicular distance from the center to the edge of the pipeline. Mean distance was compared between 1997 and 1998 with Mann–Whitney U‐test.

For Willow Flats in 1999, we compared home range sizes between animals whose home ranges were exclusively in the eastern sand hummock area (higher resource availability) and those that used any part of the western shallow soil area (lower resource availability) and also correlated home range size with SMPA as at Salt Valley.

### Kangaroo rat seed preferences and seed traits

#### Cafeteria trials

Seeds of blackbrush, Mormon tea (*Ephedra viridis*), cliffrose (*Purshia mexicana*), and rough mules ears (*Wyethia scabra*) were collected from Arches National Park, Grand Co., Utah, in 1997. Seeds of Indian ricegrass (*Achnatherum hymenoides*) were commercially obtained (Granite Seed, Lehi, Utah). To determine a preference hierarchy for the five native seed species, we conducted cafeteria‐style preference trials in two wooden arenas (area 0.5 m^2^) with hardware cloth tops and attached nest boxes. The bottom of each arena consisted of a fine screen underlain by a wooden floor constructed so that sand could be drained out, leaving seeds and seed remnants on the screen for collection. The arenas were placed outdoors at a secluded site in Arches National Park.

We live‐trapped two kangaroo rats for each trial from mixed desert shrub habitat at a distance from the study sites and fasted them 8–12 h before placing them into the arenas at dusk. We provided 4 g of each seed species to each subject in 100‐mm petri dishes placed in a randomized array with one seed species per dish. Kangaroo rats spent an entire night in the arena with access to the seeds and were released the next morning at the point of capture. We collected the remaining whole seeds (including those cached throughout the arena) and hull remnants, separated and weighed them by species, and then determined the species composition of consumed seeds by subtraction. The first six trials included Indian ricegrass, but it was eliminated because it was so highly preferred that it compromised the accuracy of estimates for the remaining species; trials with Indian ricegrass were not included in the formal analysis. Fifteen trials (seven males and eight females) were conducted in the summer of 1998 and 13 (seven males and six females) in 1999.

A compositional analysis, that is, an analysis of the relative amounts of each seed species consumed (Aebischer et al. 1993), was performed using data from the cafeteria trials using cliffrose consumption (near zero) as the denominator. The analysis used a MANOVA procedure, with year, sex, and seed species as the main factors.

#### Nutritional analysis

For each of the five native seed species used in the preference trials and for *Panicum miliaceum*, a common component of rodent laboratory diets, nutritional characteristics of the edible (i.e., hulled) portions were determined by standard proximate analysis (Fonnesbeck 1977; Van Soest 1981; Cunniff 1995; analyses performed by Agri‐Test, Inc., Twin Falls, ID). Nitrogen determined by the Kjeldahl method was multiplied by 6.25 to estimate crude protein.

To determine digestibility for seeds of these species, ten wild‐caught kangaroo rats were kept in captivity under animal care protocol #A00/01‐05 at the University of Nevada, Reno. A digestibility trial consisted of a 3‐day acclimation period and a 4‐ to 16‐day trial period. The animals received a known mass of the trial seeds (4–5 g) on each of 3 days and were monitored daily for weight and amount eaten. On day 4 and each day thereafter, the sand substrate was sifted to collect all nonsand items (feces, hulls, and uneaten seeds) and new measures of seed were given. Nonsand items were sorted and weighed to determine how much mass was consumed and how much fecal matter was produced. Cliffrose could not be tested because kangaroo rats refused to eat it in captivity. Dry matter digestibility was determined each night for each animal from the following equation (Rymer 2000):

Digestibility=(seed intake−fecal material)/seed intake

Within‐subject values were averaged before mean digestibility was calculated.

#### Palatability

Mammalian taste receptors are functionally the same across taxa (Lindemann 2001). Mammals are generally attracted to sweet tastes (Harborne 1988). As an informal test of the rank order of palatability for the five seed species included in the cafeteria trials, we asked a nonrandom sample of 50 humans (friends) to volunteer to taste‐test pairs of seeds and choose the most palatable member of each pair. Each of ten possible pairs was tested five times with the seeds presented in random order. We also interviewed participants to obtain a qualitative description of the taste of each test species.

#### Seed handling time and net energy gain

We used video trials to capture the seed handling behavior of *D. ordii* for the three most preferred seed species included in the cafeteria experiment. Animals were placed in a glass aquarium with a video camera underneath the floor. For each trial, an animal was presented with 2–3 seeds of a single species in a petri dish, and the number of frames from pickup (seed left the aquarium floor) to first hull piece dropped to last hull piece dropped onto the aquarium floor for each individual seed was subsequently counted. The video frame count data were used to calculate the mean handling time for each seed species. We then combined handling times from multiple trials with data on energy content of the seeds to calculate energy intake per unit time for each seed species.

### Field caching experiment

We tested the predictions of optimal caching theory in a caching experiment using a highly preferred seed (Indian ricegrass) and a less preferred but regularly utilized seed (blackbrush). The trials were carried out at Willow Flats from June through August 2000. Home ranges for the six kangaroo rats that took part in the caching study were calculated as described earlier (Hooge et al. 1999) by combining regular trapping data with burrow locations obtained by following animals postrelease. Locations from incidental trapping were not used in generating home ranges, so that home range calculations were independent of animal movements during the caching trials.

We observed scatterhoard placement in the field using a fluorescent powder technique that enabled us to track an animal from a bait station to its scatterhoards or larders in burrows (Breck and Jenkins 1997). Shallow trays (40 × 30 × 1.5 cm) were lined with coarse sandpaper and filled to 2 mm with fluorescent powder (Radiant Color Company, Richmond, CA). Each tray contained a single 100‐mm petri dish filled with 15 g of Indian ricegrass or blackbrush seeds. All seeds were lightly dusted with fluorescent powder in a contrasting color (blue). Kangaroo rats reliably made repeated trips to the tray until the seeds were depleted. Individuals were allowed to participate in the caching trial up to four times, as long as the trials were on different nights. The six kangaroo rats participated in 2–4 trials each. All participating animals received both seed types and all with four trials received each seed type twice. Half the individuals encountered Indian ricegrass first and half encountered blackbrush first.

Direct observation was necessary to ensure that only one animal harvested from the tray. While harvesting the seeds, the target animal would necessarily step into the fluorescent powder and then track it away. When the seeds were depleted, Sherman traps were set to identify the target animal by the presence of fluorescence on its fur. We also recorded harvesting time at the tray and time between visits to the tray.

To find scatterhoards, we followed the fluorescent trails using ultraviolet light. Scatterhoards appeared as spots of blue color, and the presence of seeds under the soil surface was confirmed by digging. Scatterhoards that had already been retrieved were identified by blue color surrounding a small depression in the soil and presence of stray seeds. These cases were included in the analysis. Fifteen of 62 scatterhoards were collected for quantification. Locations of scatterhoards were flagged and mapped the next day.

Nineteen caching trials yielded usable results, 10 with Indian ricegrass and nine with blackbrush. Three response variables were calculated: (1) mean distance to the bait tray, which measured how far the seeds were removed; (2) mean distance to the center of the cacher's home range, which measured the propensity of the cacher to remove the seeds to a place where it could likely defend them at a lower cost; and (3) mean distance to the arithmetic center of the caches, which was an index of cache dispersion.

Each response variable was tested separately in a general linear model (GLM) procedure in which seed species was the fixed main effect and individual kangaroo rat was a random blocking variable (SPSS Science 1998). The *F*‐ratio for the fixed effect was calculated with the interaction term as the denominator (Zar 1984). A similar statistical procedure was used to analyze the time that kangaroo rats spent with each seed type at the bait tray and in activities away from the tray. These tests examined independent hypotheses that were not conflicting, that is, each could be true or false independently of all the others.

To analyze the placement of caches relative to home range boundaries, we used ArcView GIS 3.2 (ESRI 1999) to visualize the cache locations layered on top of the 65% kernel home range (core area) for each cacher. Each cache was counted as either inside or outside the core area. A chi‐square heterogeneity analysis was performed to determine whether caches of each seed species were placed nonrandomly relative to home range core areas (Zar 1984). Because the 65% kernel home range is essentially a utilization distribution, the expected null ratio (caches inside:caches outside) was 0.65:0.35.

Finally, we examined whether scatterhoards were placed closer to the bait station or to the home range center of each cacher. For each trial, the distances from the arithmetic center of the caches both to the bait station and to the center of the home range were calculated. The difference between the distances was used as the response variable in a GLM model with kangaroo rat as a random factor. The model tested whether the mean difference in distance was different from zero. This procedure accounts for multiple trials per individual.

## Results

### Spatiotemporal variation in seed resources

#### Salt Valley

**Figure 2:**
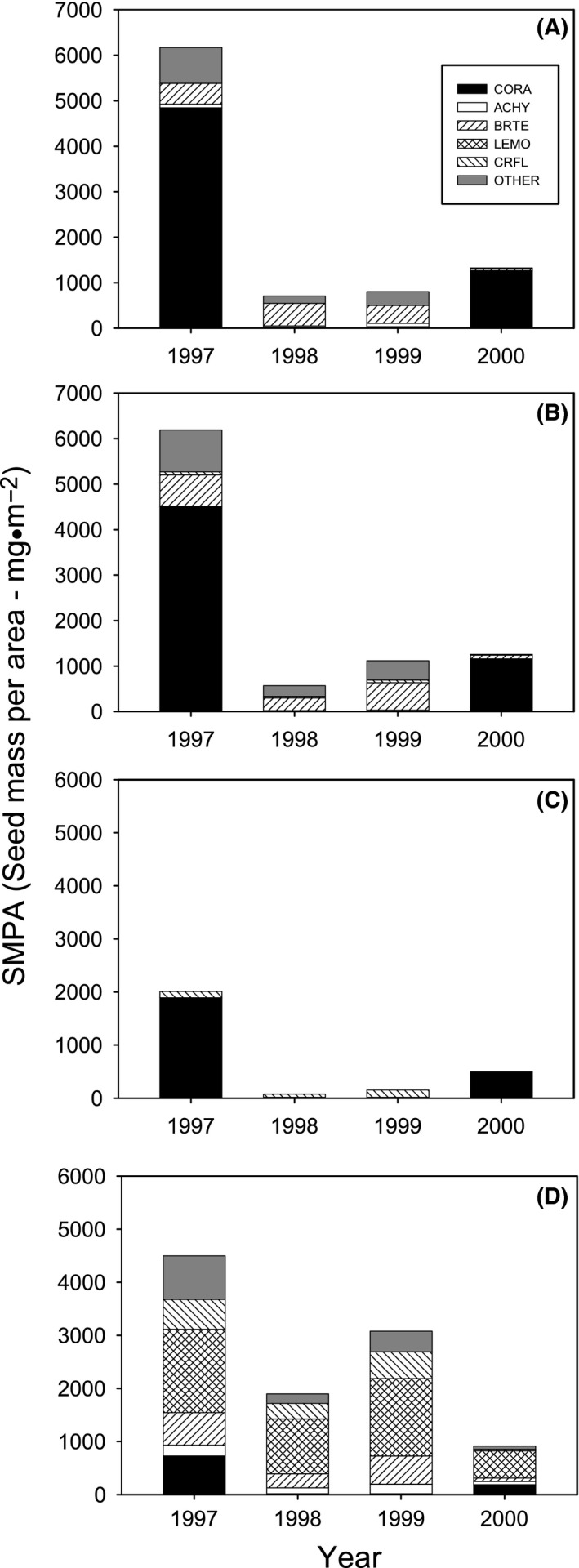
SMPA (seed mass per unit area) in (A) blackbrush, (B) wash, (C) rock, and (D) pipeline habitats at Salt Valley in each of 4 years, showing proportions contributed by each of the principal seed‐producing species (see Appendices 1, 2; Meyer and Pendleton 2015a for methods). CORA = blackbrush, ACHY = Indian ricegrass, BRTE = cheatgrass, LEMO = mountain peppergrass, CRFL = yellow catspaw. Remaining species values were pooled in OTHER.

The year 1997, with somewhat above‐average winter precipitation (Table 1), was a mast year for blackbrush at Arches National Park, and its seed rain dominated the seed resources at Salt Valley that year. It produced an estimated 79% of the seed mass in the blackbrush habitat, 73% of the seed mass in the wash habitat, and 94% of the seed mass in the rock outcrop habitat (Fig. 2). Even on the pipeline disturbance, where it was much less abundant, blackbrush accounted for 16% of the total seed mass. When these percentages are weighted by the area covered by each habitat, blackbrush seeds accounted for 79% of the total seed mass produced in 1997. Its SMPA in the blackbrush habitat was 4846 mg/m^2^, which is equivalent to 48.5 kg/ha. In 1998, a year with somewhat below‐average winter precipitation, blackbrush flowered very sparsely and did not contribute any seeds to the seed rain, a typical interannual pattern for a masting species. In the following 2 years, it produced relatively small quantities of seeds, but nonetheless contributed to the available seed resource overall (5% of total seed mass produced in 1999, a relatively wet year, and 91% of total seed mass produced in 2000, an exceptionally dry year).

Total SMPA peaked in all four habitats in 1997 (Fig. 2). The blackbrush, wash, and pipeline habitats had comparable seed production that year, while the rock outcrop habitat had an SMPA 2–3 times lower than the other habitats in this as well as other years. In the following 3 years, total SMPA was decreased by 75–95% relative to the 1997 value in the blackbrush, wash, and rock outcrop habitats. The pipeline habitat showed a much less drastic decrease in seed production in 1998 and 1999, with 2–5 times the SMPA of the blackbrush and wash habitats. Its production dropped to comparably low levels only in 2000, the driest year.

Several species in addition to blackbrush sometimes made substantial contributions to SMPA, particularly on the pipeline disturbance (Fig. 2B). Overall the second most important seed producer was the exotic annual cheatgrass (*Bromus tectorum*), which always ranked in the top three producers in the blackbrush, wash, and pipeline habitats. Mountain peppergrass (*Lepidium montanum)* was the most important seed producer on the pipeline in all 4 years. Indian ricegrass similarly made a noticeable contribution to SMPA only on the pipeline. Yellow catspaw (*Cryptantha flava*) was a notable producer in the wash and rock outcrop habitats but was especially abundant on the pipeline corridor.

#### Willow Flats

**Figure 3:**
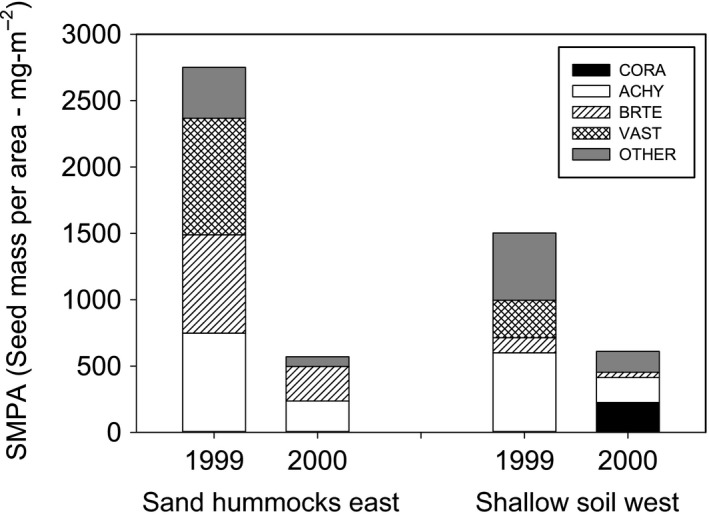
SMPA (seed mass per unit area) in two habitats at Willow Flats study site in 1999 and 2000, showing proportions of seeds contributed by each of the principal seed‐producing species. (see Appendices 1, 2; Meyer and Pendleton 2015a for methods) CORA = blackbrush, ACHY = Indian ricegrass, BRTE = cheatgrass, VAST = Vanclevea. Remaining species values were pooled in OTHER.

In 1999, total SMPA for the sand hummock habitat at Willow Flats was 2752 mg/m^2^ (Fig. 3), a value comparable to the SMPA in the pipeline habitat at Salt Valley that year (3077 mg/m^2^), and the two areas showed a similar decrease in SMPA in 2000. SMPA for the shallow soil habitat at Willow Flats was 57% of the sand hummock habitat SMPA value in 1999, but the two habitats had similarly low SMPA values in 2000. Indian ricegrass was a major producer in both habitats, as was cheatgrass. The shrub *Vanclevea stylosa* contributed substantially to SMPA in both habitats in 1999 but did not produce seeds in 2000. Blackbrush contributed to SMPA only in the shallow soil habitat and only in 2000.

### Heteromyid population response to masting

**Figure 4:**
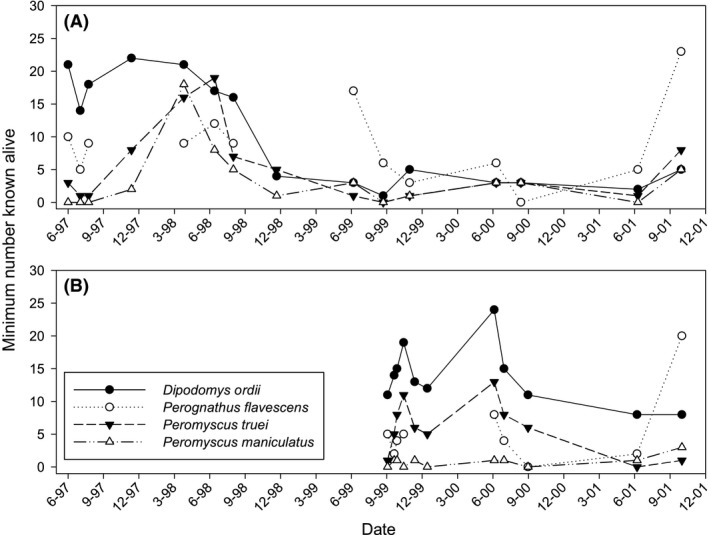
(A) Minimum number known alive (MNKA) for four granivorous rodent species: (A) at Salt Valley study site in 15 trapping sessions and (B) at Willow Flats study site in 11 trapping sessions. (winter values for hibernating *P. flavescens* not shown).

At Salt Valley, there was a pronounced trend for both heteromyids and *Peromyscus* species to peak in numbers during late 1997 through early 1998, presumably in response to the blackbrush mast event, and then to show sharp declines in August through November 1998 (Fig. 4A). Thereafter, each species remained at low numbers through June 2001 and finally showed some increase in October 2001. *Perognathus flavescens* was the exception, showing an unusual peak in June 1999, presumably a short‐term response to greater‐than‐average rainfall in the winter of 1998–1999. *Dipodomys ordii* was the most abundant species during the period of high seed abundance associated with the blackbrush mast event in 1997. Its numbers dropped sharply at the end of the first postmast summer (1998) and remained at low levels for the remainder of the study.

At Willow Flats, the four rodent species generally tracked one another closely during the 3 years of trapping (Fig. 4B). Notable population peaks occurred in October 1999 and early June 2000 when juveniles comprised 26% and 20% of the MNKA over all species. Juveniles captured in other sessions never comprised more than 4.8% of the MNKA. *Dipodomys ordii* was the most abundant species in all trapping sessions but the last, and while its numbers did decline (apparently mostly due to juvenile mortality) during the 2000 drought year, they never approached the low numbers seen at Salt Valley during the postmast years.

### Kangaroo rat–pocket mouse interactions

The observed niche overlap between *D. ordii* and *P. flavescens* for each time period at Salt Valley (Table 2) was always at the extreme lower end of the distribution of expected niche overlaps based on the randomization test (*P *=* *0.015 in 1997, *P *=* *0.009 in 1998, and *P *=* *0.007 in 1999–2000). This indicates that the two species were spatially separated within the study area on the scale of individual trap locations in each time period.

**Table 2:** Matrix of niche overlap values from captures of *D. ordii* (ord) and *P. flavescens* (flav) in the years 1997–2001 at the Salt Valley study site, Arches National Park, Utah. The data for the years 1999–2001 were combined. Values were derived using the Czekanowski index from captures at 163 trap stations where traps were considered the common resource

	ord 97	ord 98	ord 99–01	flav 97	flav 98	flav 99–01
ord 97	1	0.416	0.191	0.121	0.261	0.341
ord 98		1	0.308	0.069	0.223	0.315
ord 99–01			1	0	0.126	0.148
flav 97				1	0.197	0.080
flav 98					1	0.336
flav 99–01						1

**Figure 5:**
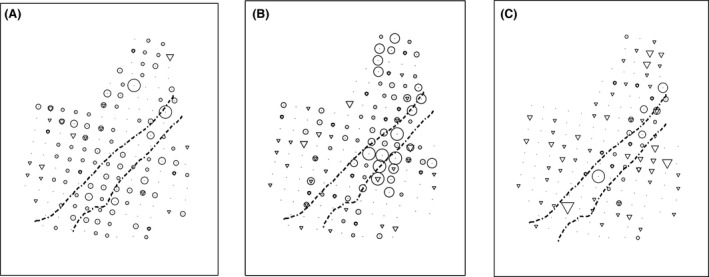
Captures of *Dipodomys ordii* (open circles) and *Perognathus flavescens* (open triangles) on the permanent trapping grid (163 traps) at Salt Valley study site in (A) 1997, (B) 1998, and (C) 1999–2001. Symbol size indicates the number of captures (1–4 and ≥5). Trap stations with no captures are shown as points. Dashed lines demarcate the pipeline corridor.

As kangaroo rats decreased in numbers through time, pocket mice used more of the space that kangaroo rats had previously occupied, that is, the between‐species niche overlap increased across years within rows in Table 2. Also, through time the pocket mice moved to traps not previously used and kangaroo rats discontinued use of some traps, so that the within‐species niche overlap decreased across years within rows. During 1997 (the mast year), kangaroo rats were well dispersed throughout the study area, whereas pocket mice were almost completely restricted to marginal areas, with very few traps used by both species (Fig. 5A). In 1998 (the first intermast year), kangaroo rat numbers were still high, but the animals tended to abandon areas dominated by blackbrush, and pocket mice began to move into these areas (Fig. 5B). Once kangaroo rat numbers dropped sharply, this species was largely absent from blackbrush‐dominated areas and pocket mice fully occupied this now‐abandoned habitat (Fig. 5C).

The odds ratio analysis showed that the two heteromyid species used the pipeline corridor differentially, and these use patterns changed across years (*G* = 3.23, df = 2, *P *=* *0.20 for the best‐fit model: all main effects and two‐way interactions). Kangaroo rats were caught more often on the pipeline corridor than pocket mice in all time periods, and in 1997, when kangaroo rats were abundant, pocket mice were never caught there (Table 3). Both species increased their use of the pipeline through time, but the increase was much greater for kangaroo rats than for pocket mice. For pocket mice, pipeline usage never exceeded that expected by chance, while for kangaroo rats in 1999–2001, pipeline usage was five times that predicted by chance. The ratio of kangaroo rat captures to pocket mouse captures decreased through time from 5.2 in 1997 to 0.47 in 1999–2001 as kangaroo rat numbers declined. Kangaroo rats tended to move onto the pipeline in years of low seed availability, and the few animals that remained in 1999–2001 used this area almost exclusively (Fig. 5B,C).

**Table 3:** Summary of captures on and off the pipeline corridor at the Salt Valley study site for the years 1997–2001. A total of 27 trapping stations were located in the pipeline disturbance area and 136 were off the pipeline

Species	Year	Captures		Odds ratio of capture on pipeline
		On pipeline	Off pipeline	
*D. ordii*	1997	35	106	1.663
	1998	57	116	2.473
	1999–2001	21	21	5.037
*P. flavescens*	1997	0	27	0
	1998	5	44	0.572
	1999–2001	13	75	0.873

### Home range size and resource abundance

At Salt Valley, there was a major between‐year difference in mean SMPA of individual home ranges (*F*_1,19_ = 150.9, *P *<* *0.001): 4486 mg/m^2^ (SE = 208) in 1997 compared to 779 mg/m^2^ (SE = 218) in 1998. Mean home range size did not differ between years, however (*t *= −1.005, df = 20, *P *=* *0.295): 4101 m^2^ (SE 489) in 1997 versus 4828 m^2^ (SE 1221) in 1998.

**Figure 6:**
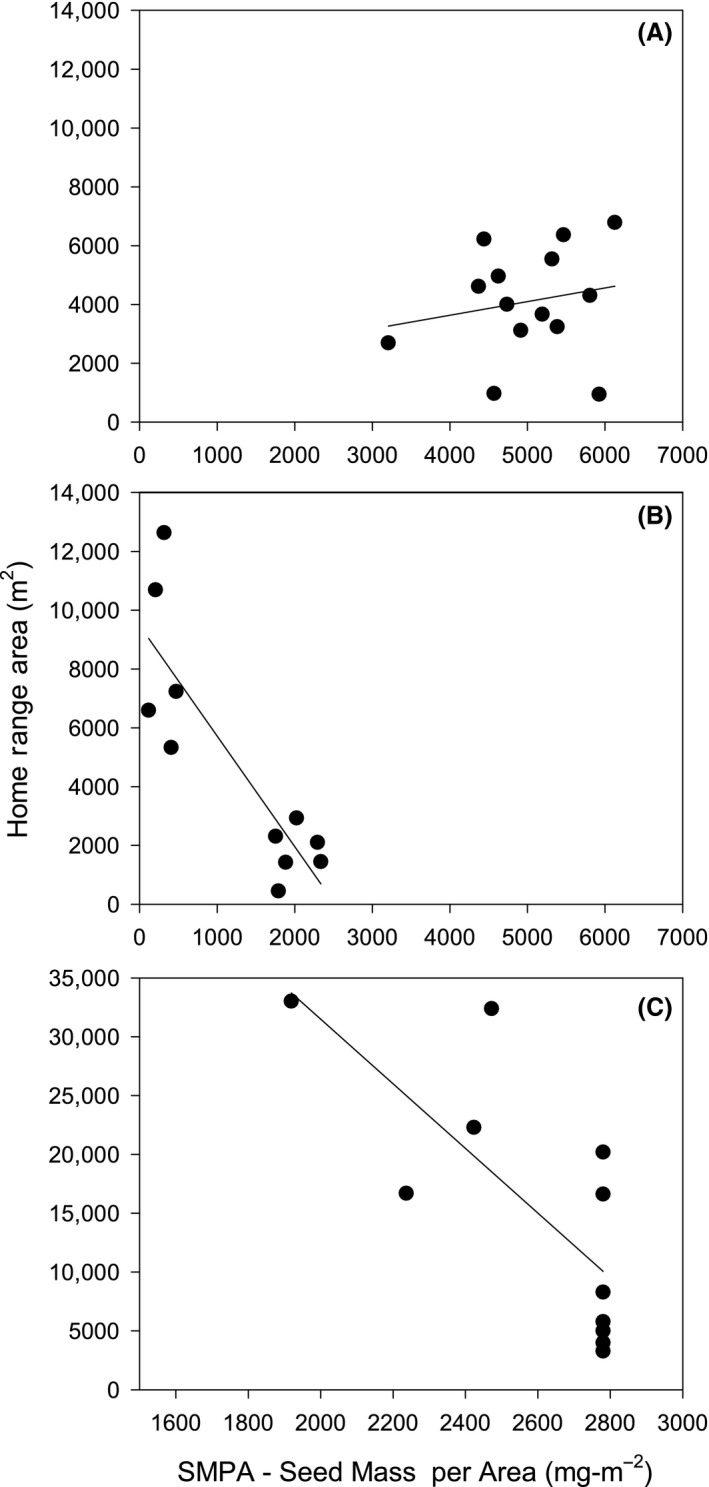
Relationship between seed mass per unit area (SMPA; mg/m^2^) and home range area at (A) Salt Valley in 1997, (B) Salt Valley in 1998, and (C) Willow Flats in 1999.

Home range SMPA was not negatively correlated with home range area at Salt Valley in 1997, a year of abundant seed resources (Pearson's *r *=* *0.194, *n *=* *14, *P *=* *0.51; Fig. 6A). However, these two variables were strongly negatively correlated in 1998, a year of limited seed resources (Pearson's *r *= −0.849, *n *=* *11, *P *=* *0.001; Fig. 6B). These results offer support for the resource abundance hypothesis by showing that animals experiencing more resource‐limited conditions increased their home range size. Centers of kangaroo rat home ranges were also closer to the pipeline corridor in 1998 than in 1997 (Mann–Whitney U = 46, *P *=* *0.037), providing more support for the prediction that kangaroo rats would move closer to the seed resource‐rich pipeline corridor in a year without blackbrush seed production.

At Willow Flats in 1999, mean home range size for animals using the shallow soil habitat averaged 23,779 m^2^ (SE 593), while mean home range size for animals using only the sand hummock habitat was 9015 m^2^ (SE 525; Mann–Whitney U = 1.00, *n*_EAST_ = 7, *n*_WEST_ = 3, *P *=* *0.03). It appeared that animals with burrows in shallow soil habitat traveled to the sand hummocks to forage. Home range SMPA at Willow Flats in 1999 was negatively correlated with home range size (Pearson's *r *= −0.740, *n *=* *10, *P *=* *0.014; Fig. 6C), providing additional support for the resource abundance hypothesis.

### Kangaroo rat seed preferences and seed traits

Kangaroo rats consumed seeds differentially in the cafeteria trials (Wilks’ lambda = 0.185, *F*_3,22_ = 32.287, *P *<* *0.0001). Indian ricegrass was highly preferred. Blackbrush and rough mules ears were both consumed more than cliffrose (*P *<* *0.0001), but Mormon tea was not, resulting in the preference ranking in Table 4. The multivariate statistic for the year effect (Wilks’ lambda = 0.678, *F*_3,22_ = 3.477, *P *=* *0.033) reflected a trend for rough mules ears to be consumed more in the second year. The effect of sex on seeds eaten (Wilks’ lambda = 0.577, *F*_3,22_ = 5.383, *P *=* *0.006) was also due to a difference in rough mules ears consumption. Females ate more rough mules ears seeds than males (*F*_1,24_ = 10.727, *P *=* *0.003), but consumption of other seeds was the same for both sexes. Males and females did not differ in body mass ($\bar{x}$_♂_ = 60.3 g, $\bar{x}$_♀_ = 57.9 g, *t*‐test, df = 24, *P *=* *0.332) or in the total amount eaten in one night ($\bar{x}$_♂_ = 2.017 g, $\bar{x}$_♀_ = 2.064 g, *t*‐test, df = 26, *P *=* *0.880).

**Table 4:** Nutritional composition (% dry mass) of five seeds native to Arches National Park, Utah, and white millet, a common species in rodent laboratory diets. Edible portions were extracted from the husks prior to analysis

Species	Preference rank	Free water	Crude fiber	Crude protein	Ash	Fat	Soluble carb.^a^	Cal/g^b^	DMD
*Panicum miliaceum*		8.9	7.6	12.8	7.8	3.5	66.2	3.73	0.943
*Achnatherum hymenoides*	1	8.7	12.8	21.0	7.5	1.5	56.4	3.52	0.884
*Wyethia scabra*	2	5.5	10.1	40.0	12.2	1.1	35.3	3.51	0.884
*Coleogyne ramosissima*	3	5.9	9.7	43.0	12.7	4.0	29.3	3.67	0.839
*Ephedra viridis*	4	6.6	15.5	31.3	9.6	2.1	41.5	3.44	0.916
*Purshia mexicana*	5	7.0	13.5	35.4	9.8	2.4	38.4	3.54	–c

DMD, dry matter digestibility for *Dipodomys ordii*.

a Nitrogen‐free extract (NFE) which represents soluble carbohydrates was figured by difference.

b Calories per gram = 4.8 (proportion of crude protein) + 9.5 (proportion of fat) + 4.2 (proportion of carbohydrate).

c *Dipodomys ordii* refused to consume seeds of this species in the laboratory.

Nutrient content of the test seeds varied widely (Table 4). Blackbrush had the highest protein content (43%), twice that of Indian ricegrass, but Indian ricegrass contained much more soluble carbohydrate (56.4% vs. 29.3%). Blackbrush also had the highest fat content (4%). There was a strong positive correlation between free water content and soluble carbohydrates (*r *=* *0.948, *P *=* *0.004). Digestibilities of all tested seeds were ≥84%.

When seeds of the five native species were presented in paired taste tests to 50 human subjects, the resulting palatability ranking was similar to the kangaroo rat preference ranking in the cafeteria trials. Seeds of Indian ricegrass and rough mules ears had a pleasant, neutral taste and were always preferred to seeds of blackbrush and Mormon tea, which in turn were emphatically preferred to seeds of cliffrose. Blackbrush had a mildly unpleasant turpentine‐like taste probably indicative of terpenoids (Robbins 1993). Cliffrose was by far the most intensely bitter and astringent. This suggests that secondary compounds affecting palatability are present in blackbrush, Mormon tea, and especially cliffrose seeds. Palatability may therefore have been an important criterion determining *D. ordii* food preferences in the cafeteria trials.

Handling time is a key component in calculating the net rate of energy gain for granivores. Blackbrush seeds had the highest energy content per gram, but hulling speed for *D. ordii* was not fast (median = 7.43 s/seed). In contrast, Indian ricegrass was fast to hull (median = 1.67 s/seed). Taking into account the different mean weights of hulled seeds (3.19 mg/seed for Indian ricegrass and 10.62 mg/seed for blackbrush) and assuming that the animals could consume the hulled seeds of different species at the same rate, the energy intake per unit time would be only slightly higher for Indian ricegrass than for blackbrush (0.36 vs. 0.33 Cal/min).

### Caching behavior and optimal caching theory

In the field caching experiment, the mean number of trips required for a kangaroo rat to harvest 15 g of seed from the bait tray was 2.8 for Indian ricegrass and 3.6 for blackbrush. A total of 62 caches were located, 29 of Indian ricegrass and 33 of blackbrush. Mean size of recovered caches was 4.87 g for Indian ricegrass and 1.25 g for blackbrush.

Kangaroo rats placed scatterhoards an average of 23.4 m (SE 2.7) away from the bait tray and 31.8 m (SE 2.9) from the center of the home range. The average index value for cache dispersion was 15.9 m (SE 1.6). There was no detectable tendency for kangaroo rats to place caches of Indian ricegrass further from the bait tray (*F*_1,4_ = 0.059, *P *>* *0.5) or to spread them out more than caches of blackbrush (*F*_1,4_ = 0.181, *P *>* *0.5). There was a trend for kangaroo rats to place Indian ricegrass seeds closer to the center of their home ranges than blackbrush seeds (*F*_1,4_ = 6.08, 0.05 < *P *<* *0.10). The difference in the mean distance from a cache to the center of the home range for the two seed species was 8.42 m. The effects of individual animals and individual by seed interaction terms were not significant (*P *>* *0.05).

There was no detectable tendency for kangaroo rats to place caches of either species within their core home range (65% home range polygon) at a greater frequency than would be predicted by chance (heterogeneity *χ* ^2^ = 0.530, df = 1, 0.25 < *P *<* *0.5). Fifty‐nine of the 62 caches were placed within the cacher's 95% polygon.

Despite the fact that blackbrush seeds are bulkier than Indian ricegrass seeds, the time spent by kangaroo rats harvesting at the tray did not differ between seed species when all individuals were included in the analysis (*F*_1,6_ = 2.51, 0.20 < *P *<* *0.50). However, only one individual did not strip the hairlike styles off the blackbrush seeds before pouching them. This animal was the major contributor to the near‐significant effect of individual nested within seed type (*F*_6,2_ = 14.66, *P *=* *0.065). Removal of this individual from the analysis showed that stripping the style makes blackbrush more time‐consuming to pouch than Indian ricegrass (*F*_1,5_ = 88.104, *P *<* *0.001). Five of six kangaroo rats were willing to invest almost twice the time (131 sec vs. 67 sec) at the bait tray to process blackbrush seeds prior to pouching them.

Individuals spent an average of 118 sec (SE = 50.9) away from the tray when caching blackbrush seeds versus 142 sec (SE = 54.0) for Indian ricegrass (*F*_1,5_ = 0.32, *P *>* *0.5); this does not support the hypothesis that kangaroo rats should spend more time optimally placing caches of the preferred species to minimize pilferage.

Kangaroo rats placed caches closer to the bait trays than to the centers of their home ranges (*F*_1,13_ = 4.84, *P *=* *0.047). They may quickly sequester seeds underground rather than immediately transporting them to areas more frequented and potentially better defended. In three trials, some of the caches were removed within 20 min after seed harvesting, presumably by the cacher before it was caught in post‐trial trapping, supporting the idea of rapid sequestration.

## Discussion

### Blackbrush mast events and heteromyid population dynamics

It is not surprising that the kangaroo rat population showed a numerical response to a masting event in blackbrush‐dominated vegetation at Salt Valley, as masting created a major seed resource that facilitated reproduction and also permitted persistence through the following year. And because blackbrush produces few seeds in the first postmast year, the kangaroo rat population could not maintain itself at the level achieved during the mast much beyond the following summer, when stored food reserves were likely exhausted. The fate of the population then depended on seed production by other species in the first postmast summer, but in areas heavily dominated by blackbrush, seed production by other species even in a favorable year would be far less than a blackbrush masting seed crop (e.g., 1999; Fig. 2).

At Willow Flats, where mixed desert vegetation produced seeds more directly in proportion to current‐year precipitation, the kangaroo rat population did not show the dramatic fluctuations observed at Salt Valley. The MNKA for kangaroo rats at Willow Flats averaged 13.6 (SE = 1.41, *n *=* *11) across all trapping sessions (1999–2001), while the MNKA at Salt Valley averaged only 3.1 (SE = 0.55, *n *=* *8) during the same period (Fig. 4).

It remains unknown whether kangaroo rat populations in blackbrush‐dominated landscapes rebound to mast year population highs via local reproduction or via immigration from more diversely vegetated source areas like Willow Flats. Jones (1993) suggested that *Dipodomys* rarely disperse more than 100 m from their natal sites and that juveniles occupy open spaces among resident adults. Low‐density populations in extensive monospecific stands of blackbrush may thus be especially vulnerable to local extinction during nonmast years.

### Interference competition between kangaroo rats and pocket mice

We found evidence that kangaroo rats (body mass 50–65 g) exert behavioral dominance over the much smaller pocket mice (body mass 7–9 g) and competitively exclude them from resource‐rich areas through interference competition (Reichman and Price 1993). The very low between‐species niche overlap within years meant that the two heteromyid species did not use the same areas as much as predicted by chance, and this was evident in the spatial representation of the trapping data (Fig. 5). In the mast year for blackbrush, when kangaroo rats were at maximum abundance, no pocket mice were ever captured in the kangaroo rat‐preferred habitat of the pipeline corridor. In addition, kangaroo rats were dispersed throughout the blackbrush‐dominated area during the mast year, and very few trap locations were used by both species. As MNKA decreased to ≤5 for kangaroo rats in the postmast period from late 1998 through 1999–2001, remaining individuals occupied the pipeline corridor almost exclusively, even though it comprised only 15% of the available area of the trapping grid. We observed greater capture numbers and more evenly dispersed capture locations of pocket mice in blackbrush‐dominated areas that had been vacated by kangaroo rats. This is strong evidence that the two species compete and that interference may be the mechanism for exclusion of pocket mice from resource‐rich areas. Because of their smaller size, pocket mice may be better able than kangaroo rats to subsist on the meager seed resources in blackbrush‐dominated areas during intermast periods. Pocket mice at Salt Valley successfully produced offspring during the intermast period even though they were largely restricted to blackbrush‐dominated habitat (Figs. 4a, 5). They did not experience a steep reduction in MNKA until the driest year (2000) and rebounded rapidly when conditions improved in 2001.

### Blackbrush mast events and the resource abundance hypothesis

The major contribution of blackbrush to the seed rain during the mast event in 1997 allowed for an uncoupling of kangaroo rat home range size from SMPA, as home ranges of all sizes had abundant summer food. Ord's kangaroo rats eat about 2.5 g of hulled seed material (ca. 4 g of intact seeds) per day (J. Auger, unpublished data). All home ranges in summer 1997 contained at least four times the absolute amount of seeds needed for a kangaroo rat to survive for three seasons (274 days), that is, until renewed seed availability in spring. In 1998, 4 of 11 home ranges contained inadequate new seed production to sustain a kangaroo rat for the same time period. Under these limiting conditions, home range size was negatively correlated with resource density.

This study is one of the few to demonstrate a negative relationship between resource density and home range size in an unmanipulated system rather than through artificial resource addition, which may have methodological problems caused either by an influx of animals in response to resource addition in unfenced experiments or by artifacts of fencing in fenced experiments (Orland and Kelt 2007; Prevedello et al. 2013). The results of this natural experiment provided clear support for the resource abundance hypothesis (Akbar and Gorman 1993; Lacher and Mares 1996).

Home ranges at Salt Valley did not differ in size between years. One explanation is that animals in highly productive years may range farther to search out patches where seed harvest rate is most efficient (Adams 2001; Steury and Murray 2003). The returns would offset the additional travel cost and predation risk, and home ranges would be expected to contain more total resources than necessary to meet essential metabolic needs.

The movement of kangaroo rat home range centers closer to the pipeline corridor at Salt Valley in 1998 also supports the idea that home range placement is driven by food resources. That summer, newly produced seeds most available to kangaroo rats were those of species common in the disturbed soils of the pipeline corridor. In postmast time periods, the pipeline corridor had greater seed production than blackbrush habitat, which accounts for the increasing odds ratios of captures there for both species.

Disturbed areas such as the pipeline in Salt Valley may reduce the chance of local kangaroo rat extinction by providing more food resources than blackbrush‐dominated vegetation during nonmast years and by concentrating adult individuals so that they are more likely to find a mate when conditions improve. Even though it comprised only a small portion of the study area, its shape (a belt traversing diagonally through the study site; Fig. 5) and consistently high seed production over the 2‐year period of the home range study resulted in use by 70% of the kangaroo rats on the study site in both years.

### Kangaroo rat seed preferences and seed traits

Nutritional characteristics of native seeds found at Salt Valley in Arches National Park showed no clear relationship with kangaroo rat rank preference in this study. Food preferences could not be explained by simple maximizing rules, but instead seemed to represent a complex integration of palatability, net energy gain, nutrient gain, and water balance criteria.

A simple explanation for the strong preference for Indian ricegrass exhibited by kangaroo rats in this and numerous other studies (Kelrick and MacMahon 1985; Longland et al. 2001; Veech 2001) is that its seeds contain >50% of soluble carbohydrates. It would therefore be the best choice among the native seeds in this study to maximize metabolic water intake (Frank 1988). Evidence that kangaroo rats were sensitive to water yield of seeds in this study is provided by the fact that palatable, protein‐rich rough mules ears seeds were consumed more in a year of above‐average precipitation (1999). Because protein digestion is water expensive (Frank 1988), the higher consumption of protein‐rich seeds in a less water‐limited year makes biological sense. Rough mules ears was also consumed more by females, whose protein needs are greater during seasons of reproduction (Robbins 1993), but the same was not true for blackbrush.

Indian ricegrass seeds lack secondary compounds, as do many seeds preferred by heteromyids (Henderson 1990). Astringency and bitterness are often indicators of secondary compounds, and the least preferred seeds in the cafeteria trials (Mormon tea and cliffrose) were rejected apparently on that basis. It may be advantageous for a plant species whose seeds are dispersed by seed predators to have somewhat unpalatable seeds, as this may increase the chances that seeds will be cached rather than consumed immediately (Vander Wall 2010; Wang et al. 2013). Also, palatability of seeds with secondary compounds may improve after long‐term storage in scatterhoards (W. S. Longland, personal communication; Dearing 1997).

Of the assemblage of seeds in this study, blackbrush seeds are perhaps ecologically the most important food for heteromyids at Arches National Park. They had a higher protein and lipid content than the more preferred seeds of Indian ricegrass but a lower carbohydrate and free water content. The seeds of blackbrush had a mildly unpleasant taste to humans, but were readily consumed by kangaroo rats both in the field and in the laboratory. They required a bigger investment in husking time per seed but yielded an energy return per unit time comparable to that of Indian ricegrass because of their larger size and higher lipid content. Their high protein content could also be an important factor in diet quality in this nitrogen‐limited desert ecosystem (Henderson 1990). These data support the idea that heteromyids could subsist exclusively on blackbrush seeds for extended periods and receive adequate nutrition. Because blackbrush is likely consumed as the staple food primarily during autumn and winter following a mast year, its disadvantages as a water source may be relatively unimportant.

### Caching experiment and optimal caching theory

In the field caching experiment, there was little evidence that kangaroo rats differentially cached seeds in accordance with their preferences in cafeteria trials as predicted by optimal caching theory. Seeds were taken relatively short distances from the source, and kangaroo rats did not place caches of the preferred Indian ricegrass seeds farther from the source tray or spread them out more than blackbrush caches, although there was a trend to place Indian ricegrass caches closer to home range centers. By these measures, investment of energy in caching decisions was similar for the two seed types.

One possible explanation for this apparent lack of differential investment is that seeds of the two species may have been valued more or less equivalently by kangaroo rats in the field despite being eaten differentially in cafeteria trials, which require the subject to choose what it will eat in the present. Caching behaviors presumably evolved because delaying consumption of an item for options in future consumption was advantageous (Vander Wall 1990). Kangaroo rats were willing to spend twice as much time at the seed trays to process blackbrush seeds before pouching them, indicating that those seeds were indeed valued in relation to the effort required to harvest and store them. Measurement of preference for animals that store food to survive seasonal periods of deficit should perhaps be based on choices made in the field throughout the year. In field studies of kangaroo rat diet based on stomach contents, Indian ricegrass comprised over 50% of food consumed during the early growing season, whereas shrub seeds supplemented the diet to a greater extent later in the year (Henderson 1990; Longland et al. 2001).

Another possible explanation for the lack of differential investment in caching the two seed species is that the powder‐tracking technique reveals only the first decision of the caching individual. Scatterhoarding granivores manage their caches intensively (Hirsch et al. 2013). They may do this to solidify their memory of cache locations, monitor food quality, or reduce apparency of the caches to pilferers (Vander Wall and Jenkins 2003). Consequently, seed movements are highly dynamic (Vander Wall 1995). When a heteromyid forages in a rich patch such as a seed tray, it may elect to focus its efforts on maximal collection rather than optimal storage, that is, the cost of losing the highly apparent food to competitors may outweigh the cost of losing hastily made caches to pilferers before the cacher can return to move them to less vulnerable places. This behavior was described by Jenkins and Peters (1992) as rapid sequestering and identified by Jenkins et al. (1995) as a likely explanation of cache placement patterns by *D. merriami* in the laboratory. Despite the relatively low numbers of kangaroo rats at Willow Flats, there were competitors present at 42% of bait stations. The rapid sequestering hypothesis is consistent with our results, specifically the short return times to the bait stations (<2 min), the placement of caches in locations not optimal for long‐term storage (i.e., closer to bait stations than to areas of nonoverlapping home ranges), and the observation of animals quickly removing caches after first sequestering the seeds. Kangaroo rats in this study did behave to reduce the risk of cache loss by usually making more than one cache per harvesting trip and by spreading out the caches at >2 m spacing, a distance that substantially reduces the risk of pilferage (Daly et al. 1992).

## Conclusions

In this study in desert vegetation dominated by a masting desert shrub, we showed that both spatial and temporal aspects of the population dynamics of Ord's kangaroo rat, its primary seed predator and disperser, were strongly and directly impacted by dramatic interannual differences in seed resource availability caused by the masting phenomenon. The kangaroo rat population declined to low levels after the first postmast summer, when stored seed reserves had apparently been depleted. This pattern was not seen in nearby mixed desert vegetation dominated by nonmasting species, where kangaroo rat numbers remained relatively high and constant across years. We showed that competitive interactions between kangaroo rats and the smaller resident pocket mice were strongly mediated by this temporal and spatial variability in seed resources. Home range size in blackbrush‐dominated habitat was negatively correlated with seed resource density in the seed‐limited postmast year but not in the mast year. We found evidence that a pipeline disturbance acted as a refugium for kangaroo rats in years of low resource availability in blackbrush‐dominated habitat because it supported plant species with more consistent annual seed production. We also determined that blackbrush seeds were ecologically the most important seed resource for kangaroo rats in areas where blackbrush was the dominant species. Its seeds could meet kangaroo rat nutritional requirements and were a heavily used and valued resource even though not highly preferred for immediate consumption.

## Conflict of Interest

None declared.

## Appendix 1 Vegetation composition at Salt Valley and Willow Flats study sites

**Table A1.1:** Density (plants/m^2^), frequency, and cover of plant species in four habitats at Salt Valley study site, Arches National Park. Density determined for perennials; cover determined for shrubs only. (CORA = pure stands of blackbrush, WASH = sandy washes, ROCK = rocky areas with shallow, patchy soils, PIPE = pipeline disturbance with deeper soils)

Species	CORA			WASH			ROCK			PIPE		
	Density	% Freq.	% Cover	Density	% Freq.	% Cover	Density	% Freq.	% Cover	Density	% Freq.	% Cover
Trees, shrubs, and succulents												
*Artemisia filifolia*	0	0	0	0.23	16.7	4.9	0	0	–	0.02	1.7	1.2
*Coleogyne ramosissima*	1.85	83.3	29.3	0.42	28.3	6.8	2.10	82.5	14.6	0.20	20.0	5.5
*Ephedra torreyana*	0.01	0.8	0	0.03	1.7	1.4	0.03	2.5	1.0	0	0	0
*Ephedra viridis*	0.84	27.5	3.5	0.70	23.3	5.6	0	0	0	0.17	6.7	3.1
*Ericameria nauseosa*	0	0	0	0.03	1.7	–	0	0	0	0	0	0
*Eriogonum microthecum*	0	0	0	0	0	0	0.38	20.0	0	0	0	0
*Gutierrezia sarothrae*	0.025	1.7	0	0.83	41.7	3.7	0.08	2.5	0.1	1.20	55.0	3.3
*Juniperus osteosperma*	0	0	0	0	0	2.5	0	0	4.5	0	0	0
*Opuntia erinacea*	0.30	27.5	1.8	0.12	10.0	0.7	0	0	0	0.05	5.0	0
*Petradoria pumila*	0	0	0	0	0	0	1.18	42.5	1.4	0	0	0
*Purshia mexicana*	0	0	0	0.03	3.3	5.3	0	0	0	0	0	0
*Quercus havardii*	0.05	1.7	0	0	0	0	0	0	0	0	0	0
*Yucca harrimannii*	0	0	0	0.03	3.3	0.1	0	0	0	0	0	0
Herbaceous perennials												
*Abronia fragrans*	0	0	–	0.13	5.0	–	0	0	–	0.12	5.0	–
*Achnatherum hymenoides*	0.11	10.0	–	0.08	5.0	–	0.025	2.5	–	0.28	6.7	–
*Ambrosia acanthicarpa*	0	0	–	0.03	5.0	–	0	0	–	0.30	20.0	–
*Arenaria fendleri*	0	0	–	0	0	–	0.05	5.0	–	0	0	–
*Aristida purpurea*	0	0	–	0	0	–	0	0	–	0.15	6.7	–
*Astragalus lentiginosus*	0	0	–	0	0	–	0.10	5.0	–	0	0	–
*Cryptantha flava*	0.03	2.5	–	0.12	10.0	–	0.28	22.5	–	1.03	41.7	–
*Euphorbia fendleri*	0	0	–	0.03	1.7	–	0.025	20.0	–	0	0	–
*Pleuraphis jamesii*	0.08	1.7	–	0.38	16.7	–	0.18	7.5	–	0.28	6.7	–
*Sphaeralcea parvifolia*	0	0	–	0.03	3.3	–	0	0	–	0.40	26.7	–
*Sporobolus flexuosus*	0.02	0.8	–	0	0	–	0	0	–	0	0	–
*Streptanthus cordatus*	0	0	–	0	0	–	0.025	2.5	–	0	0	–
*Townsendia incana*	0.02	1.7	–	0.07	1.7	–	0	0	–	0.07	10.0	–
*Wyethia scabra*	0.07	3.3	–	0	0	–	0	0	–	0	0	–
Annuals												
*Bromus tectorum*	–	65.8	–	–	65.0	–	–	10.0	–	–	93.3	–
*Chaenactis stevioides*	–	0.8	–	–	0	–	–	0	–	–	0	–
*Cryptantha crassisepala*	–	10.0	–	–	6.7	–	–	0	–	–	20.0	–
*Erodium cicutarium*	–	0	–	–	1.7	–	–	0	–	–	0	–
*Euphorbia parryi*	–	0	–	–	5.0	–	–	0	–	–	10.0	–
*Gilia gunnisonii*	–	1.7	–	–	6.7	–	–	0	–	–	10.0	–
*Helianthus petiolaris*	–	0	–	–	1.7	–	–	0	–	–	0	–
*Lepidium lasiocarpum*	–	1.7	–	–	3.3	–	–	0	–	–	10.0	–
*Machaeranthera tanacetifolia*	–	0	–	–	0	–	–	0	–	–	5.0	–
*Phacelia ivesiana*	—	5.0	–	–	6.7	–	–	0	–	–	0	–
*Plantago patagonica*	—	9.2	–	–	3.3	–	–	0	–	–	1.7	–
*Streptanthella longirostris*	–	5.8	–	–	5.0	–	–	2.5	–	–	18.3	–
*Vulpia octoflora*	–	34.2	–	–	8.3	–	–	0	–	–	16.7	–

**Table A1.2:** Density (plants/m^2^, frequency, and cover of plant species in the sandy hummock habitat on the east side and the shallow cryptogamic soil habitat on the west side of Willow Flats study site, Arches National Park. Density was determined for perennials; cover was determined only for shrubs

Species	Sandy hummocks			Shallow cryptogamic soil		
	Density	% Freq	% Cover	Density	% Freq	% Cover
Trees, shrubs, and succulents						
*Artemisia filifolia*	0.14	11	3.08	0.01	1	1.16
*Atriplex canescens*	0.01	1	0.12	0	0	0
*Coleogyne ramosissima*	0.02	1	0.68	0.32	22	5.32
*Ephedra viridis*	0.21	7	0.22	0.22	7	0.48
*Ephedra torreyana*	0.01	1	0	0	0	0
*Eriogonum leptocladon*	0.22	18	3.00	0.06	6	0
*Gutierrezia sarothrae*	0.24	10	0.48	0.18	10	0.20
*Juniperus osteosperma*	0.02	2	0.12	0.02	2	1.04
*Opuntia erinacea*	0.01	1	0	0	0	0.08
*Poliomintha incana*	0.16	9	1.28	0.20	11	2.12
*Quercus havardii*	0.26	9	4.88	0.68	20	4.90
*Vanclevea stylosa*	0.31	22	3.68	0.10	6	1.72
*Yucca harrimannii*	0	0	0.56	0	0	0
Herbaceous perennials						
*Abronia fragrans*	0.02	1	–	0	0	–
*Achnatherum hymenoides*	1.12	46	–	0.81	41	–
*Aristida purpurea*	0.04	3	–	0.15	8	–
*Asclepias macrosperma*	0.01	0	–	–	1	–
*Astragalus ceramicus*	0.69	13	–	3.11	32	–
*Astragalus* sp.	0.03	2	–	0.08	6	–
*Cryptantha flava*	0.19	15	–	0.15	14	–
*Heliotropium convolvulaceum*	0	0	–	0.01	1	–
*Hymenopappus filifolius*	0	0	–	0.05	5	–
*Muhlenbergia pungens*	0.34	16	–	0.51	17	–
*Orobanche* sp.	0.01	1	–	0	0	–
*Psoralea lanceolata*	0.26	15	–	0.50	22	–
*Senecio multilobatus*	0.01	1	–	0.01	1	–
*Sporobolus giganteus*	0.05	5	–	0	0	–
*Sporobolus flexuosus*	0.25	17	–	0.07	7	–
*Thelesperma subnudum*	0.02	2	–	0	0	–
*Townsendia incana*	0	0	–	0.01	2	–
Annuals						
*Bromus tectorum*	–	62	–	–	22	–
*Camissonia* sp.	–	5	–	–	0	–
*Cryptantha crassisepala*	–	10	–	–	3	–
*Dicorea canescens*	–	1	–	–	0	–
*Erigeron bellidiastrum*	–	12	–	–	1	–
*Eriogonum* sp.	–	9	–	–	2	–
*Euphorbia parryi*	–	25	–	–	3	–
*Gilia gunnisonii*	–	17	–	–	10	–
*Machaeranthera tanacetifolia*	–	18	–	–	5	–
*Mentzelia* sp.	–	1	–	–	0	–
*Plantago patagonica*	–	4	–	–	0	–
*Salsola iberica*	–	13	–	–	0	–
*Streptanthella longirostris*	–	0	–	–	2	–
*Vulpia octoflora*	–	61	–	–	68	–

## Appendix 2 Estimating seed production

See Meyer and Pendleton (2015a) for a detailed description of seed production quantification for blackbrush. The description below pertains to all species except blackbrush.

All seeds were cleaned from the air‐dried plant matter by hand‐screening to obtain near 100% purity and weighed to obtain mg seeds/plant. A subsample of seeds from each collection was then incubated on blotter paper at 15/25°C (12 h:12 h) after suitable chilling and scored weekly for 4 weeks for germination. Total viability percentage was determined by adding the germination percentage to the filled ungerminated percentage (determined by a cut test). Mean mg viable seed/plant was then calculated by multiplying the mean seed mass per plant by the proportion of viable seeds. This correction was necessary because only filled seeds provide food for heteromyids.

For most species, there was a year during the study when seed production was very low or zero, providing a second point for constructing the linear relationship that allowed us to estimate seed production for each species in years when no seed collections were made. Multiplying the estimated mean viable seed mass per plant for each year by plant density in each habitat yielded an estimate of SMPA (mg/m^2^) for each species. For this calculation, annuals were assigned densities of 5 plants/m^2^ for low abundance category, 30 plants/m^2^ for medium abundance, and 75 plants/m^2^ for high abundance (midpoints of category ranges). This method of estimating seed production assumed that filled and unfilled seeds were the same weight, that seed size and viability were the same between years regardless of precipitation, that individuals sampled were representative of the species over all habitats, and that perennial plant density did not change across years. For annuals, among‐year differences in density were implicitly included in the reproductive output estimates because estimated lower production in drier years was due to lower density as well as smaller plant size.

Species with seeds below the size threshold at which kangaroo rats cease significant harvest (0.25 mg; Price and Reichman 1987), or that produced seeds in the autumn at Salt Valley, after home range parameters were determined, were not included in the SMPA (seed mass per unit area, mg/m^2^) calculations but are included on the table for species for which data are available.

**Table A2.1:** Seed production data for species other than blackbrush^a^ whose seeds are a potential food resource for heteromyid rodents at Salt Valley and Willow Flats, Arches National Park, Utah. Lifespan is given as perennial (P), biennial (B), or annual (A)

Species	Seed mass (mg/seed)	% Filled	Viable seed (mg/plant)	Life span	Date collected
*Abronia fragrans*	1.31	99	107	P	May 2001
*Achnatherum hymenoides*	3.19	29	568	P	Jun 2000
*Ambrosia acanthicarpa*	7.62	89	727	A	Oct 1999
*Aristida purpurea*	2.24	59^2^	366	P	Jun 2000
*Artemisia filifolia*	0.22	81	1848	P	Nov 1999
*Astragalus ceramicus*	11.39	10	12	P	May 2001
*Bromus tectorum*	2.09	100	42	A	May 2001
*Cryptantha flava*	1.91	81	50	P	Jun 2000
*Cymopterus newberryi*	6.68	43	334	P	May 2001
*Ephedra viridis*	22.46	–	–	P	Oct 1999
*Euphorbia parryi*	0.65	64	46	A	Oct 1999
*Gilia gunnisonii*	0.34	84	32	A	Oct 1999
*Gutierrezia sarothrae*	0.50	100	575	P	Oct 1999
*Hesperostipa comata*	10.51	93	–	P	May 2001
*Lepidium montanum*	0.39	87	602	P	Jun 2000
*Lupinus brevicaulis*	12.10	48	95	A	May 2001
*Machaeranthera tanacetifolia*	0.30	53	199	A	Oct 1999
*Muhlenbergia pungens*	0.24	81	10	P	Oct 1999
*Opuntia erinacea*	27.6	85	–	P	Jun 2000
*Pleuraphis jamseii*	1.69	–	–	P	Oct 1999
*Purshia mexicana*	8.36	20	402	P	Jun 2000
*Sphaeralcea parvifolia*	1.18	84	1392	P	Jun 2000
*Sporobolus flexuosus*	0.12	99	808	P	Oct 1999
*Hesperostipa comata*	10.51	93	–	P	May 2001
*Streptanthella longirostrus*	0.38	35	201	A	May 2001
*Vanclevea stylosa*	0.92	46	2835	P	Oct 1999
*Vulpia octoflora*	0.51	87	22	A	May 2001
*Wyethia scabra*	12.33	5	–	P	Jun 2000

a Seed production data for blackbrush (*Coleogyne ramosissima*) were determined by a different method described in the text (see Meyer and Pendleton 2015a for details).
